# In Situ Real-Time Chemiluminescence Imaging of Reactive Oxygen Species Formation from Cardiomyocytes

**DOI:** 10.1155/2008/941729

**Published:** 2009-02-25

**Authors:** Yunbo Li, Haiou Shen, Hong Zhu, Michael A. Trush, Ming Jiang, Ge Wang

**Affiliations:** ^1^Division of Biomedical Sciences, Edward Via Virginia College of Osteopathic Medicine, Blacksburg, VA 24060, USA; ^2^Department of Biomedical Sciences and Pathobiology, Virginia-Maryland Regional College of Veterinary Medicine, Virginia Tech, Blacksburg, VA 24061, USA; ^3^Biomedical Imaging Division, Virginia Tech-Wake Forest University School of Biomedical Engineering and Sciences, Blacksburg, VA 24060, USA; ^4^Department of Environmental Health Sciences, The Johns Hopkins University Bloomberg School of Public Health, Baltimore, MD 21205, USA

## Abstract

We have applied the highly sensitive chemiluminescence (CL) imaging technique to investigate the in situ ROS formation in cultured monolayers of rat H9c2 cardiomyocytes. Photon emission was detected via an innovative imaging system after incubation of H9c2 cells in culture with luminol and horseradish peroxidase (HRP), suggesting constitutive formation of ROS by the cardiomyocytes. Addition of benzo(a)pyrene-1,6-quinone (BPQ) to cultured H9c2 cells resulted in a 4-5-fold increase in the formation of ROS, as detected by the CL imaging. Both constitutive and BPQ-stimulated CL responses in cultured H9c2 cells were sustained for up to 1 hour. The CL responses were completely abolished in the presence of superoxide dismutase and catalase, suggesting the primary involvement of superoxide and hydrogen peroxide (H_2_O_2_). In contrast to BPQ-mediated redox cycling, blockage of mitochondrial electron transport chain by either antimycin A or rotenone exerted marginal effects on the ROS formation by cultured H9c2 cells. Upregulation of cellular antioxidants for detoxifying both superoxide and H_2_O_2_ by 3*H*-1,2-dithiole-3-thione resulted in marked inhibition of both constitutive and BPQ-augmented ROS formation in cultured H9c2 cells. Taken together, we demonstrate the sensitive detection of ROS by CL imaging in cultured cardiomyocytes.

## 1. INTRODUCTION

Reactive oxygen species (ROS) have been implicated in the pathogenesis of various human diseases, particular cardiac disorders [1, 2]. ROS include a number of oxygen-containing reactive species, among which superoxide and hydrogen peroxide (H_2_O_2_) are most widely studied in biomedical field [3, 4]. ROS attack cellular constitutes, including lipids, proteins, and nucleic acids, leading to oxidative stress and tissue injury. Due to the detrimental effects of ROS, mammalian cells have evolved a number of antioxidants to protect cells from oxidative injury. However, under pathophysiological conditions, ROS formation may overwhelm the normal cellular antioxidant defenses, and as such oxidative tissue injury and disease process occur [3, 4].

There are a number of cellular sources of ROS formation, which include mitochondrial electron transport chain (ETC), NAD(P)H oxidase, uncoupled nitric oxide synthases, and cytochrome P450 enzymes [3, 4]. The above cellular sources of ROS formation are implicated in oxidative stress of various tissues, including vasculature and myocardium [1, 2]. Because of the multiple cellular component nature of mammalian tissues, detection of cell-specific formation of ROS is of critical importance for understanding the oxidative mechanisms of disease process and for developing antioxidative stress-based strategies for intervention of oxidative tissue degeneration. In this context, over the last several decades various methods have been developed for detecting ROS formation in biological systems. These include the electron paramagnetic resonance (EPR) spin trapping, luminescent methods, as well as a number of biochemical assays [1, 5, 6]. While EPR spin trapping technique is of the highest specificity for detecting oxygen radicals, its sensitivity is limited [5, 6]. Moreover, EPR technique and most of the biochemical assays for detecting ROS are unable to directly visualize the ROS formation in a highly sensitive real-time manner. Direct sensitive in situ detection of ROS formation from cultured cells provides important insight into the molecular and biochemical mechanisms by which ROS mediate pathophysiological processes. Accordingly, in this study, we have applied the highly sensitive chemiluminescence (CL) imaging technique to investigate the in situ ROS formation in cultured monolayers of rat H9c2 cardiomyocytes and the factors that modulate the ROS generation in this widely used in vitro model for cardiac cell physiology and pathophysiology.

## 2. MATERIALS AND METHODS

### 2.1. Chemical and reagents

D3T with a purity of 99.8% was generously provided by Dr. Mary Tanga at SRI International (Menlo Park, Calif, USA) and Dr. Linda Brady at the National Institute of Mental Health (Bethesda, Md, USA). Benzo(a)pyrene-1,6-quinone (BPQ) was from Midwest Research Institute (Kansas City, Mo, USA). Dulbecco's modified Eagle's medium (DMEM), penicillin, streptomycin, and fetal bovine serum (FBS) were from Gibco-Invitrogen (Carlsbad, Calif, USA). All other chemicals and reagents were from Sigma-Aldrich (St. Louis, Mo, USA).

### 2.2. Cell culture and treatment

Rat H9c2 cardiomyocytes (ATCC, Manassas, Va, USA) were cultured in DMEM supplemented with 10% FBS, 100 U/mL of penicillin, and 100 *μ*g/mL of streptomycin in tissue culture flasks at 37°C in a humidified atmosphere of 5% CO_2_. The cells were fed every 2-3 days, and subcultured once they reached 70–80% confluence. For CL imaging experiments, 2 × 10^5^ cells were plated in each well of 6-well plates. The CL images were acquired directly with the confluent cells (2.8 × 10^5^ cells/well or 0.3 × 10^5^/cm^2^) in culture at 37°C after washing of the cells with phosphate buffered saline (PBS). To initiate the CL response, 10 *μ*M luminol and 5 *μ*g/mL horseradish peroxidase (HRP) were added to the cell monolayers in 6-well plates, with each well containing 2 mL PBS. For experiments on induction of antioxidants, H9c2 cells were incubated with 100 *μ*M D3T dissolved in dimethyl sulfoxide (DMSO; 0.1% final concentration) in culture medium for 48 hours. Control cells received 0.1% DMSO only.

### 2.3. Cell extract preparation

H9c2 cells were collected and resuspended in ice-cold 50 mM potassium phosphate buffer, pH 7.4, containing 2 mM EDTA and 0.1% Triton X-100. The cells were sonicated, followed by centrifugation at 13 000 g for 10 minutes at 4°C. The resulting supernatants were collected, and the protein concentrations were quantified with Bio-Rad protein assay dye (Hercules, Calif, USA) using bovine serum albumin as the standard. The samples were kept on ice for measurement of the antioxidants as described below.

### 2.4. Measurement of cellular antioxidants

Cellular superoxide dismutase (SOD) activity was determined by the method of Spitz and Oberley, as described before [7, 8]. The cellular SOD activity was calculated using a concurrently run Cu,ZnSOD (Sigma-Aldrich) standard curve, and expressed as units per mg of cellular protein.

Cellular NAD(P)H:quinone oxidoreductase 1 (NQO1) activity was determined using dichloroindophenol (DCIP) as the two-electron acceptor and NADPH as the electron donor in the presence or absence of dicumarol, as described before [7]. The dicumarol-sensitive NQO1 activity was calculated using the extinction coefficient of 21.0 mM^−1^cm^−1^, and expressed as nmol of DCIP reduced per minute per mg of cellular protein.

Cellular catalase activity was determined by measuring the decomposition of H_2_O_2_ at 240 nm, according to the method of Aebi [9], and expressed as *μ*mol of H_2_O_2_ consumed per minute per mg of cellular protein.

Cellular GSH content was determined by measuring the formation of the fluorescent conjugate from reaction of GSH with *o*-phthalaldehyde according to the procedure described previously [7]. Cellular GSH content was calculated using a concurrently run GSH (Sigma-Aldrich) standard curve, and expressed as nmol of GSH per mg of cellular protein.

Cellular glutathione reductase (GR) activity was measured according to the method initially reported by Wheeler et al. [10] with modifications, as described previously [9]. GR activity was calculated using the extinction coefficient of 6.22 mM^−1^cm^−1^, and expressed as nmol of NADPH consumed per minute per mg of cellular protein.

Cellular glutathione peroxidase (GPx) activity was determined by the method of Flohé and Günzler [11] with slight modifications, as described before [12]. This assay is based on the formation of GSSG from GPx-catalyzed oxidation of GSH by H_2_O_2_, coupled with NADPH consumption in the presence of exogenously added GR. GPx activity was calculated using the extinction coefficient of 6.22 mM^−1^cm^−1^, and expressed as nmol of NADPH consumed per minute per mg of cellular protein.

### 2.5. In situ real-time CL imaging

**Figure 1:**
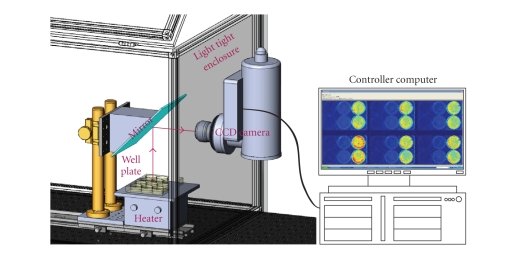
Diagram depicting the highly sensitive chemiluminescence imaging system.

As shown in Figure 1, a liquid nitrogen cooled back-illuminated charge-coupled device (CCD) camera (VersArray 2048B, Princeton Instruments, Trenton, NJ, USA) with a Nikon 50 mm f/1.2 lens was used to image the 6-well cell culture plates. The CCD sensor has a 27.6 × 27.6 mm imaging area with 2048 × 2048 pixels. It has a >80% quantum efficiency for visible spectrum range. To capture the ultra low light image, a hardware binning of 8 × 8 was selected to increase the sensitivity by 64 times. To reduce the noise, the CCD sensor was cooled to −110°C, and a 50 kHz read out frequency was used to minimize the dark current and read out noise. The culture plates were placed on a heated plate (37°C). An enhanced aluminum front mirror was placed above the culture plates to redirect the photons to the camera. To ensure that the imaging experiment environment is totally dark, the imaging system is placed in a light tight enclosure. After placing the culture plates in the imaging system, we immediately took 6 CL images, each with ten minutes of exposure time.

Before we extracted out the data, we calibrated the intensity of the CCD sensor by using dark frames to remove the background and thermal noise in a totally dark environment. Each dark frame was collected with the same integral time at the same cooling temperature in the experiment. A master dark frame was then computed by averaging 20 dark frames. For each CL image, we subtracted the master dark frame from it to get the final image. For each well in the culture plates, we summed the pixel value inside the well and divided it by the number of pixels in the well to get an average intensity for each well.

### 2.6. Statistical analysis

Data are expressed as means ±SEM from at least 3 separate experiments unless indicated otherwise. Differences between 2 groups were analyzed by Student's *t*-test. Statistical significance was considered at *P* < .05.

## 3. RESULTS

### 3.1. Detection of basal and BPQ-stimulated ROS formation by CL imaging in cultured monolayers of H9c2 cells

**Figure 2:**
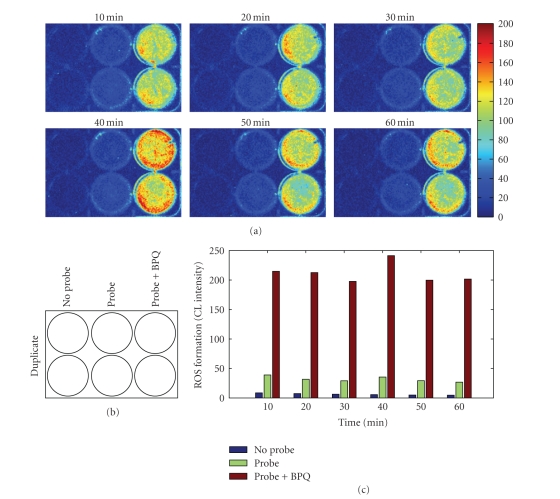
In situ real-time CL imaging of basal and BPQ-stimulated ROS formation in cultured monolayers of H9c2 cardiomyocytes. H9c2 cells were cultured in 6-well plates. Immediately prior to CL imaging, confluent cells in culture were washed once with PBS followed by addition of 2 mL PBS containing 1 *μ*M BPQ or other reagents, as described under Materials and Methods section. (a) Representative CL images acquired at the indicated time points; the images for the first 10 minutes were acquired with a 5-minute delay; (b) layout of treatment groups; probe refers to luminol/HRP; (c) quantification of time-dependent ROS formation by luminol/HRP-amplified CL imaging. Data in (c) represent averages of two measurements.

**Figure 3:**
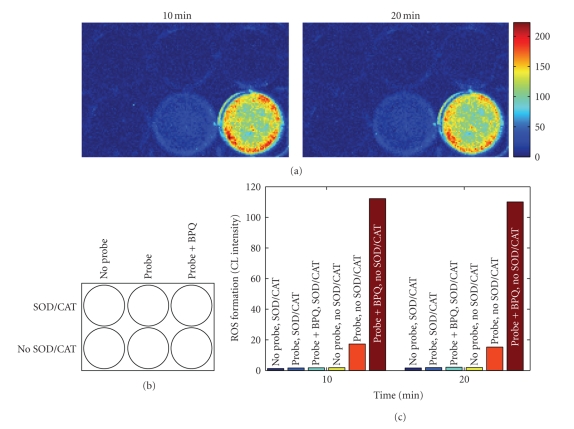
Effects of exogenously added SOD/CAT on basal and BPQ-stimulated ROS formation in cultured monolayers of H9c2 cardiomyocytes. The experimental condition was the same as that described in the legend of Figure 2 except that SOD (250 units/mL) and CAT (250 units/mL) were added to the top 3 wells. (c) Shows the quantitative data of the images in (a).

As shown in Figure 2, incubation of cultured monolayers of H9c2 cells with luminol/HRP led to CL responses as detected by the highly sensitive imaging system (see Figure 1), indicating that H9c2 cells in culture could constitutively release ROS. Notably, no CL responses were elicited by adding luminol/HRP to the plate wells containing PBS alone (data not shown). In addition, under our experimental conditions, viability of H9c2 cells in cultures was >99% based on trypan blue exclusion assay (data not shown). Dramatically augmented CL responses were observed after addition of 1 *μ*M BPQ, a mitochondrial redox cycler, to the cells in culture, and the CL responses remained elevated for up to 1 hour. The intensity of the BPQ-stimulated CL responses was 4-5 times that of basal CL responses (see Figure 2). Both constitutive and BPQ-induced CL responses were completely abolished in the presence of exogenously added SOD (250 units/mL) and catalase (250 units/mL) (see Figure 3).

### 3.2. Detection by CL imaging of the effects of mitochondrial ETC inhibitors on ROS formation in cultured monolayers of H9c2 cells

**Figure 4:**
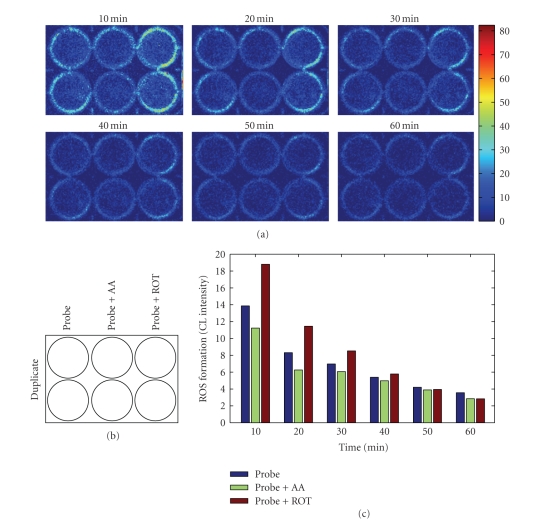
In situ real-time CL imaging of ROS formation in cultured H9c2 cardiomyocytes exposed to inhibitors of mitochondrial ETC. H9c2 cells were cultured in 6-well plates. Immediately prior to CL imaging, confluent cells in culture were washed once with PBS followed by addition of 2 mL PBS containing 10 *μ*M AA or ROT in the presence of probe (luminol/HRP), as described under Materials and Methods section. (a) Representative CL images acquired at the indicated time points; (b) layout of treatment groups, probe refers to luminol/HRP, (c) quantification of time-dependent ROS formation by luminol/HRP-amplified CL imaging. (c) Shows the quantitative data of the images in (a).

To further investigate the involvement of mitochondria in cellular ROS release, H9c2 cells in culture were exposed to two commonly used mitochondrial inhibitors, antimycin A (AA) and rotenone (ROT). As shown in Figure 4, AA treatment slightly decreased the CL responses, whereas exposure to ROT led to small increases in the CL responses. The marginal effects by either AA or ROT on the CL responses were only obvious during the first 30 minutes of treatment. From 30 to 60 minutes, the CL responses among the 3 treatment groups were essentially the same (see Figure 4). AA and ROT were dissolved in DMSO (0.1%). Control cells received 0.1% DMSO only.

### 3.3. Induction of cellular antioxidants by D3T and the effects on basal and BPQ-stimulated ROS formation in cultured monolayers of H9c2 cells

**Figure 5:**
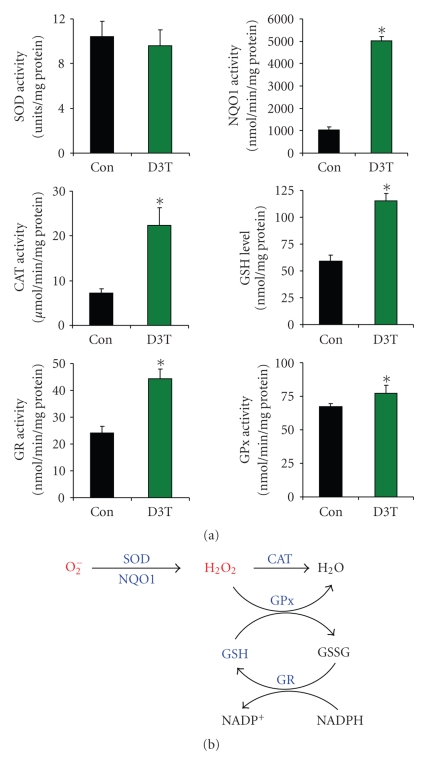
Induction of endogenous antioxidants by D3T in cultured H9c2 cardiomyocytes and schematic illustration of ROS detoxification by antioxidants. (a) H9c2 cells were incubated with 100 *μ*M D3T in culture medium for 48 hours followed by measurement of the indicated antioxidants, as described under Materials and Methods section. Data represent mean ±SEM (*n* = 3-4).∗: significantly different from control; (b) detoxification of superoxide and H_2_O_2_ by various cellular antioxidants.

**Figure 6:**
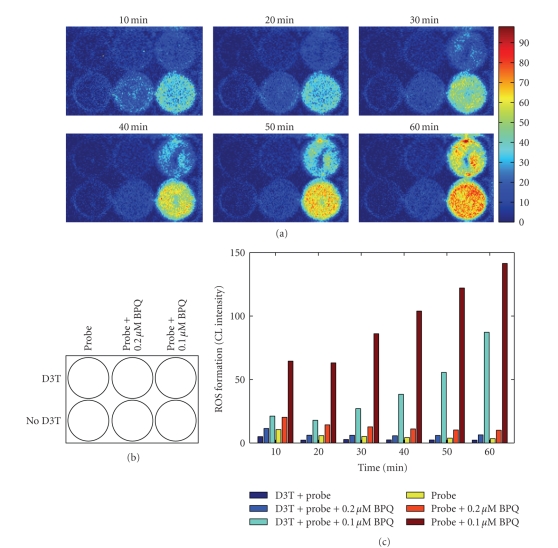
In situ real-time imaging of the effects of D3T pretreatment on basal and BPQ-stimulated ROS formation in cultured monolayers of H9c2 cardiomyocytes. H9c2 cells were treated with or without 100 *μ*M D3T for 48 hours in culture medium before CL imaging experiment. For CL imaging, confluent cells in culture were washed once with PBS followed by addition of 2 mL PBS containing 0.2 and 1 *μ*M BPQ or other reagents, as described under Materials and Methods section. (a) Representative CL images acquired at the indicated time points; (b) layout of treatment groups, probe refers to luminol/HRP, (c) quantification of time-dependent ROS formation by luminol/HRP-amplified CL imaging. Data in (c) represent averages of two measurements.

As shown in Figure 5, incubation of H9c2 cells with 100 *μ*M D3T for 48 hours resulted in significant induction of cellular NQO1, CAT, GSH, GR, and GPx. These antioxidants are crucially involved in detoxification of ROS, including superoxide and H_2_O_2_ [3]. To determine if upregulation of cellular antioxidants by D3T could blunt ROS formation by H9c2 cells, the cardiomyocytes in culture were first treated with D3T and then exposed to BPQ. As shown in Figure 6, D3T pretreatment resulted in marked inhibition of BPQ-stimulated ROS formation. Notably, D3T pretreatment also dramatically decreased the constitutive formation of ROS by cultured H9c2 cells. The inhibitory effects of D3T pretreatment on both constitutive and BPQ-stimulated ROS formation were sustained during the 1-hour period of experiment (see Figure 6). Also as shown in Figure 6, in contrast to 1 *μ*M BPQ, 0.2 *μ*M BPQ-elicited CL responses decreased over 60 minutes in untreated cell cultures. 

## 4. DISCUSSION

Although ROS have been extensively implicated in the pathogenesis of cardiac disorders, studies on direct in situ CL imaging of ROS formation in cultured cardiac cells are lacking in the literature. In this study, we have applied a highly sensitive CL imaging system (see Figure 1) to investigate the in situ real-time ROS formation in cultured monolayers of rat H9c2 cardiomyocytes, a widely used in vitro cell model for studying cardiac cell biology and cytoprotection [13, 14]. By using this innovative CL imaging system, we have demonstrated that significant amounts of ROS could be released from H9c2 cells in culture under unstimulated conditions (see Figure 2). While the exact cellular sources for this constitutive ROS formation in H9c2 cells remain to be elucidated, one possible site could be the mitochondrial ETC. In this regard, mitochondria account for roughly 40–50% of the total mass of cardiomyocytes. Considering the high mitochondrial activity in cardiomyocytes, we next determined if BPQ could stimulate the ROS formation from H9c2 cells in culture. Indeed, dramatically augmented CL responses were observed after adding 1 *μ*M, and even 0.2 *μ*M BPQ to the cultured monolayers of H9c2 cells (see Figures 2 and 6), suggesting that BPQ is a potent ROS generator in cardiomyocytes. It remains unclear why the CL responses elicited by 0.2 *μ*M BPQ decreased over 60 minutes in untreated cell cultures (see Figure 6). It might be due to the significant detoxification of the small amount of BPQ (0.4 nmol/2.8 × 10^5^ cells) in H9c2 cells, leading to decreased availability of the free BPQ molecules that undergo redox cycling to produce ROS.

BPQ is a quinone metabolite derived from benzo(a)pyrene, an environmental pollutant implicated in cardiovascular diseases [15, 16]. Benzo(a)pyrene is also present in cigarette smoking, which is a major risk factor for human cardiovascular diseases [15, 17]. Metabolism of benzo(a)pyrene by mammalian tissues, including cardiovascular tissue, can form significant amounts of BPQ [18]. We have previously demonstrated that BPQ preferentially undergoes redox cycling in mitochondria, leading to ROS formation [6, unpublished observation]. Thus, the mitochondrial ETC could also be the site where BPQ underwent redox cycling to generate ROS in the cultured monolayers of H9c2 cells. To further investigate the involvement of mitochondrial ETC in ROS formation by H9c2 cells, AA and ROT were used to selectively block the electron transport at complexes I and III, respectively [19]. The marginal effects of either AA or ROS on ROS formation by H9c2 cells (see Figure 4) suggested that blocking the mitochondrial ETC was not an effective mechanism for altering ROS formation by H9c2 cells. The redox cycling of BPQ thus appears to be a much more effective pathway for augmentation of ROS formation by cardiomyocytes. This is an important observation considering the involvement of benzo(a)pyrene in cardiovascular diseases. The potent and sustained stimulation of ROS formation by BPQ in cardiomyocytes would create significant oxidative stress in myocardium. In this context, augmented ROS formation and the subsequent oxidative cell injury in myocardium are crucial events underlying cardiac disorders [1, 2]. Likewise, inhibition or scavenging of ROS in myocardium has been shown to be an effective strategy for intervention of oxidative cardiac injury [20, 21].

In this study, we also demonstrated that pretreatment with D3T significantly upregulated cellular antioxidants and markedly inhibited both constitutive and BPQ-stimulated ROS formation in cultured monolayers of H9c2 cells (see Figure 6). D3T is a cruciferous sulfur-containing compound, which potently induces antioxidants in mammalian cells/tissues [22]. In H9c2 cells, D3T potently upregulated NQO1, CAT, GSH, GR, and GPX (see Figure 5). NOQ1 is recently found to scavenge superoxide [23]. CAT, GSH, GR, and GPx are key cellular factors primarily for detoxification of H_2_O_2_ [3]. As illustrated in Figure 5(b), coordinated induction of the above cellular antioxidants in H9c2 cells would enhance detoxification of both superoxide and H_2_O_2_, and thereby leading to the decreases in luminol/HRP-amplified CL responses. This is the first study demonstrating that upregulation of cellular antioxidants by D3T was accompanied by markedly decreased oxidative stress induced by BPQ in cultured cardiomyocytes. The above finding also implicated D3T as a promising chemoprotective nutraceutical for intervention of oxidative cardiac injury associated with exposure to benzo(a)pyrene as well as other pollutants that give rise to redox-cycling quinones. In this regard, consumption of cruciferous vegetables rich in organosulfur compounds, including dithiolethiones, is associated with decreased incidence of cardiovascular events [24, 25].

In summary, by using the highly sensitive CL imaging system, we demonstrate that cultured monolayers of H9c2 cardiomyocytes constitutively release ROS under physiological conditions, and the redox cycler BPQ potently stimulates the ROS formation in the cultured cardiomyocytes. With this innovative imaging system, we also demonstrate that upregulation of cellular antioxidants by cruciferous D3T appears to be a highly effective strategy for mitigating oxidative stress induced by BPQ in the cultured cardiomyocytes. These observations lay a basis for investigation of myocardial oxidative stress as well as cardioprotection in animal models. Although most of the data presented in this work were from 2 independent measurements, our ongoing research on luminol/HRP-based CL imaging further confirms the ability of this innovative technique to reproducibly detect ROS in both cellular systems and in vivo animals.

## ABBREVIATIONS

AA: Antimycin A
BPQ: Benzo(a)pyrene-1,6-quinone
CAT: Catalase
CL: Chemiluminescence
D3T: 3*H*-1,2-dithiole-3-thione
DCIP: Dichloroindophenol
EPR: Electron paramagnetic resonance
ETC: Electron transport chain
FBS: Fetal bovine serum
GPx: Glutathione peroxidase
GR: Glutathione reductase
GSH: Reduced form of glutathione
H_2_O_2_: Hydrogen peroxide
HRP: Horseradish peroxidase
NQO1: NAD(P)H:quinone oxidoreductase 1
PBS: Phosphate buffered saline
ROS: Reactive oxygen species
ROT: Rotenone
SOD: Superoxide dismutase.
